# Givenness hierarchy theoretic sequencing of robot task instructions

**DOI:** 10.3389/frobt.2025.1640535

**Published:** 2025-09-24

**Authors:** Zhao Han, Daniel Hammer, Kevin Spevak, Mark Higger, Aaron Fanganello, Neil T. Dantam, Tom Williams

**Affiliations:** Department of Computer Science, Colorado School of Mines, Golden, CO, United States

**Keywords:** givenness hierarchy, document planning, natural-language generation, anaphora generation, collaborative robotics

## Abstract

**Introduction:**

When collaborative robots teach human teammates new tasks, they must carefully determine the order to explain different parts of the task. In robotics, this problem is especially challenging, due to the situated and dynamic nature of robot task instruction.

**Method:**

In this work, we consider how robots can leverage the Givenness Hierarchy to “think ahead” about the objects they must refer to so that they can sequence object references to form a coherent, easy-to-follow series of instructions.

**Results and discussion:**

Our experimental results (n = 82) show that robots using this GH-informed planner generate instructions that are more natural, fluent, understandable, and intelligent, less workload demanding, and that can be more efficiently completed.

## 1 Introduction

Robots in domains ranging from collaborative manufacturing to intelligent tutoring will need to use sequences of utterances to teach or otherwise provide information to human interlocutors. In collaborative manufacturing, for example, a robot may need to instruct a worker as to how to perform a complex task over several steps. In intelligent tutoring, a robot may need to instruct a child as to how to procedurally solve a mathematics problem. In these types of domains, substantial flexibility exists in the set of instructions that the robot can convey, and the order in which instructions are given.

In the natural language generation (NLG) community, this task of determining the overarching structure of multiple sentences of generated language is referred to as *document planning*. As the name suggests, however, most previous approaches to document planning are designed for non-situated, purely textual domains. Situated dialogues like robot task instructions, in contrast, require speakers to take into account the evolving context in which described objects change both physically and in the minds of their interlocutors and those dialogues play out in real-time. One underexplored facet of interaction contexts that is critical for robot task instruction is interlocutors’ cognitive context. When interactants engage in situated interactants, they jointly manipulate the *cognitive status* that objects have within their dialogue–whether those objects are in focus, activated, familiar, and so forth–a concept formalized by Gundel et al. (1993) in their classic Givenness Hierarchy. This manipulation of cognitive status critically shapes subsequent dialogue moves, as cognitive status determines the *referring forms* that can be felicitously used. An object may only be referred to as “it”, for example, if the speaker believes the object is *in focus* for their interlocutor, whereas *the* $\left\langle N^{\prime }\right\rangle$ may be used if the speaker believes the listener can uniquely identify the object based on description $\left\langle N^{\prime }\right\rangle$ . The Givenness Hierarchy has been validated across many disparate natural languages (Gundel et al., 2010).

Enabling robots to similarly leverage this theory to effectively leverage more concise and coherent referring forms (e.g., anaphoric and deictic references) could facilitate a number of benefits (Arnold and Zerkle, 2019), as these types of referring forms make dialogue more efficient (and thus less costly to listen to) (Tily and Piantadosi, 2009), more predictable (and thus cognitively easier to follow and more humanlike) (Williams and Arnold, 2019), and more conforming to Gricean conversational maxims of cooperative speech (Grice, 1975). Additionally, the impact of these effects are magnified in situated contexts (Levinson, 2004).

We believe that leveraging these benefits in robotics will thus further improve task performance and user satisfaction, similar to the advantages gained through shorter object descriptions by Wallbridge et al. (2021), who showed that shorter object descriptions improved human task performance and were also preferred by their test subjects. Furthermore, enabling robots to leverage more concise referring forms could reduce interactants’ cognitive workload (cf. Han et al., 2021), which, when too high, degrades human performance (Xie and Salvendy, 2000). Human-like, context-dependent referring forms have been shown to reduce workload (Campana et al., 2011), and high-cognitive-status referents are conducive to the use of such forms. Moreover, a high working memory load slows spoken-word recognition time (Hadar et al., 2016). This strain on language processing could be ameliorated through more concise referring forms. With this theoretical position, we thus argue that language-enabled robots should pay close attention to how those instructions shape and are shaped by cognitive status dynamics.

In this work, we present the results of a human-robot interaction study (N = 82) that evaluates the performance of a cognitive-status informed approach to robot task instruction sequencing originally presented by Spevak et al. (2022), compared to a baseline with an uninformed classical planner. A Pepper robot instructed participants to finish two tasks, electrical fan assembly and school supply sorting, We measured objective performance (speed and accuracy of following the instructions) and subjective perceptions (naturalness, fluency, intelligence, and workload).

## 2 Background and related work

Our approach is fundamentally grounded in the psycholinguistic theory of the Givenness Hierarchy (GH) (Gundel et al., 1993). The GH is comprised of a hierarchical set of six tiers of cognitive statuses *{in focus* ⊆ *activated* ⊆ *familiar* ⊆ *uniquely identifiable* ⊆ *referential* ⊆ *type identifiable}*, each of which is associated with a set of referring (or pronominal) forms, and wherein each higher status encompasses all lower statuses (Gundel et al., 1993):

1. ‘In Focus’: An entity is considered ‘In Focus’ if it is singularly at the center of attention. ‘In Focus’ entities can be referred to with the referring form ‘It’.
2. ‘Activated’: An entity is considered ‘Activated’ if it has a representation in working memory. ‘Activated’ entities can be referred to singularly as ‘that’, ‘this’, or ‘this [N]’ where ‘[N]’ is a noun-phrase description of the entity.
3. ‘Familiar’:An entity is considered ‘Familiar’ if it has a representation in long-term memory. ‘Familiar’ entities can be referred to singularly as ‘that [N]’ where ‘[N]’ is a noun-phrase description of the entity.
4. ‘Uniquely Identifiable’: An entity is considered ‘Uniquely Identifiable’ if a unique instance of the entity can be described. ‘Uniquely Identifiable’ entities can be referred to singularly as ‘the [N]’, where ‘[N]’ is a noun-phrase description of the entity.
5. ‘Referential’: An entity is considered ‘Referential’ if a new representation of the entity can be created from a description of the entity. ‘Referential’ entities can be referred to singularly with the indefinite ‘this [N]’ where ‘[N]’ is a noun-phrase description of the entity.
6. ‘Type Identifiable’: An entity is considered ‘Type Identifiable’ if the type of entity can be referred to. ‘Type Identifiable’ entities can be referred to as ‘a [N]’, where ‘[N]’ is a noun-phrase description of the type of entity.

For a reference to an entity to be appropriate, the entity’s cognitive status must be equal or greater status to the referring form. For example, an object that is *in focus* can be referred to with the pronoun “it”. Furthermore, a speaker using “it” implicitly signals a belief that the object is *in focus* in the mind of the listener. Similarly, when “that $\left\langle N^{\prime }\right\rangle$ ” is used, the listener can infer that the entity is at least familiar, but may also be activated or even in focus.

The GH has been used for both natural language understanding (Williams et al., 2016; Williams and Scheutz, 2019; Kehler, 2000; Chai et al., 2004) and natural language generation (Pal et al., 2020) in robotics. For natural language understanding, the GH is applied to solve the problem of mapping a linguistic reference to a referent, allowing a robot to receive a command of “hand it to me” and decipher what entity “it” refers to. A variety of work leveraging the GH for this purpose (Kehler, 2000; Chai et al., 2004; Williams et al., 2016; Williams and Scheutz, 2019) and typically uses GH to justify data structures and algorithms that facilitate the resolution of anaphora and other natural language references.

For natural language generation, the GH has been mainly applied to referring form selection (Pal et al., 2020; 2021; Han et al., 2022). Pal et al. (2020) first used Bayesian filters to model cognitive status. Then they used a set of situated features, such as physical distance, to train a computational model of referring form selection using the explainable decision tree algorithm (Pal et al., 2021). Notably, Pal et al. (2020), Pal et al. (2021) used a situated referring dataset collected by Bennett et al. (2017) from a dyadic human-human task where pairs of participants collaboratively re-arranged an environment. Later, Han et al. (2022) introduced a partially observable environment (Han and Williams, 2022) to collect more repeated, invisible objects to achieve a richer set of cognitive statuses.

While the aforementioned approaches advance natural language understanding (NLU) to facilitate natural language generation (NLG), a key task in NLG is referring expression generation (REG) (Krahmer and Van Deemter, 2012; Reiter and Dale, 1997): determining how to refer to an entity to disambiguate from a set of distractors. REG is a classic NLG problem, with traditional algorithms like the Incremental Algorithm (Reiter and Dale, 1997) still enjoying widespread success. In the past decade, there has also been significant recent work on facilitating REG in situated domains. Fang (Roy, 2002; Fang et al., 2013; Meo et al., 2014; Zarrieß and Schlangen, 2016; Williams and Scheutz, 2017; Pal et al., 2021; Han et al., 2022) presented an approach that considers interactant’s knowledge of and preference for entities in a shared environment. There have also been algorithms for referring to objects in visual scenes (Roy, 2002; Meo et al., 2014; Zarrieß and Schlangen, 2016), although these do not account for references to non-visible objects. Recently, HRI researchers have presented approaches grounded in robot architectures, like DIST-PIA (Williams and Scheutz, 2017), which extends the Incremental Algorithm (Reiter and Dale, 1997) for use in uncertain domains, and which uses a consultant framework to manage distributed, heterogeneous knowledge sources. Most recently, GAIA (Higger and Williams, 2024) similarly extends the Incremental Algorithm under a Givenness Hierarchy theoretic framework, using GH-theoretic cognitive status to restrict the set of distractors that must be eliminated.

In this work, we focus on using GH for a key NLG task not considered in prior work. Modular NLG pipelines (Reiter, 2000) typically include modules for sentence planning (deciding how to communicate a sentiment), referring expression generation (selecting properties to use to refer to referents), and linguistic realization (ensuring grammatical correctness (Gatt and Krahmer, 2018)). Above all these components sits the document planner, which decides on an overarching sequence of sentiments to communicate in order to achieve a larger communicative goal (McDonald, 1993).

In our previous work (Spevak et al., 2022), we presented the first approach to GH-theoretic approach to situated document planning, which incorporates the GH-theoretic cognitive status of a robot’s human interlocutor to generate optimal document plans. We encode cognitive status as constraints in mixed integer programming (MIP) and integrate the constraints into an MIP formulation of classical planning. In contrast, our GH-informed planning (Spevak et al., 2022) incorporates an example objective function that rewards high-cognitive-status referents. This approach enables the generation of document plans (in this use case, robot task instruction sequences) with high inter-sentential coherence, and facilitates effective use of anaphora over definite descriptions (e.g., “it” over “the N”). The system offers a proof-of-concept for the use of cognitive status as state variables in planning and optimization approaches for NLG.

Overall, this approach seeks to enable more effective document planning by leveraging cognitive status. Our key insight is that failing to account for cognitive status may harm inter-sentential coherence. For instance, these approaches may introduce more referents than strictly needed (or repeatedly re-introduce referents), requiring full definite descriptions rather than shorter anaphoric phrases. In contrast, an approach that aims to use and continue referring to task-relevant entities that are already in focus or activated could lead to greater inter-sentential coherence, shorter and easier-to-follow dialogues, and perhaps even fundamentally simpler plans overall. In this work, we seek to scientifically demonstrate these benefits through a human-subject experiment.

## 3 Materials and methods

### 3.1 Hypotheses

Based on our initial validation, we believe that a GH-informed planner considering the cognitive statuses of objects in interlocutors’ minds will lead to object reuse and sub-task separation. Thus, its produced instructions will be easier to follow, leading us to formulate seven key hypotheses. When robots’ task instructions are generated by a GH-informed document planner, as compared to a classical planner, we hypothesize that (H1) more people will successfully finish the task (H1.1) and at each step (H1.2); (H2) people will finish tasks faster (H2.1), measured by task completion time, and finish each step faster (H2.2), measured by instruction completion time; (H3) instructions will be perceived as more natural, (H4) more fluent, and (H5) more understandable; (H6) the robot will be perceived as more intelligent, and (H7) will impose less cognitive load. To test these hypotheses, we conducted a human-subjects experiment.

### 3.2 Task design

To compare the Cognitive Status (CS)-informed document planner with a classical planner baseline, we designed two collaborative assembly tasks that Pepper could instruct a human to finish. We formalized the robot’s task of generating instructions as a Situated Document Planning domain called *assembly*. It has three general manipulation actions that a robot can instruct a human, i.e., *put in*, *take out*, and *attach*: putting an object into or taking an object out of a container, and attaching two objects together.

**FIGURE 1:**
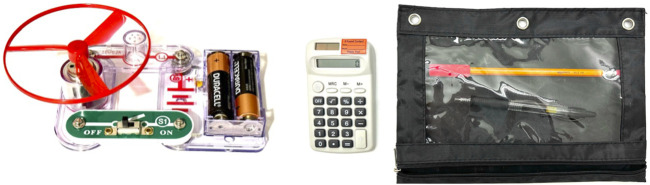
The fully-assembled electrical fan and sorted school supplies that participants were asked to finish.

Within the assembly domain, the two tasks (Figure 1) we designed were *electrical fan assembly* (hereby called the “Hard Task”) and *school supply sorting* (hereby called the “Easy Task”). The first, “hard” task involves the assembly of an electric fan using an Elenco Snap Circuits Junior kit. The required parts are a propeller, a motor, a lamp, a switch, a battery, a battery case, a battery box, and a propeller case. Figure 1 shows the final fan product on the left. The second column of Table 1 shows the planner output for this task. The kit allows for many configurations, so subjects need to understand and follow the robot’s instructions in order to correctly assemble it. However, the assembly itself is quite easy as this kit is designed as a children’s toy. The task is expected to be moderately intuitive to the subjects, as it uses widely familiar objects such as batteries, but also requires actions that subjects likely have no experience with (i.e., connecting proprietary Snap Circuits Junior parts).

**TABLE 1:** Electrical fan assembly task plan and instructions.

Planner type	Planner output	Surface realization with cognitive statuses
Classical	(take-out batteries battery-box)	*“Take the batteries (U) out of the battery box (U)”*
	(attach motor lamp)	*“Attach the motor (U) to the lamp (U)”*
	(put-in batteries battery-case)	*“Put those (A) in the battery case (U)”*
	(attach lamp switch)	*“Attach that (A) to the switch (U)”*
	(take-out propeller propeller-case)	*“Take the propeller (U) out of the propeller case (U)”*
	(attach switch battery-case)	*“Attach that (A) to that battery case (F)”*
	(attach propeller motor)	*“Attach that (A) to that motor (F)”*
GH-Informed	(take-out batteries battery-box)	*“Take the batteries (U) out of the battery box (U)”*
	(put-in batteries battery-case)	*“Put them (I) in the battery case (U)”*
	(attach switch battery-case)	*“Attach the switch to that (A)”*
	(attach lamp switch)	*“Attach the lamp (U) to it (I)”*
	(attach motor lamp)	*“Attach the motor to it (I)”*
	(take-out propeller propeller-case)	*“Take the propeller (U) out of the propeller case (U)”*
	(attach propeller motor)	*“Attach it (I) to that motor (F)”*

**FIGURE 2:**
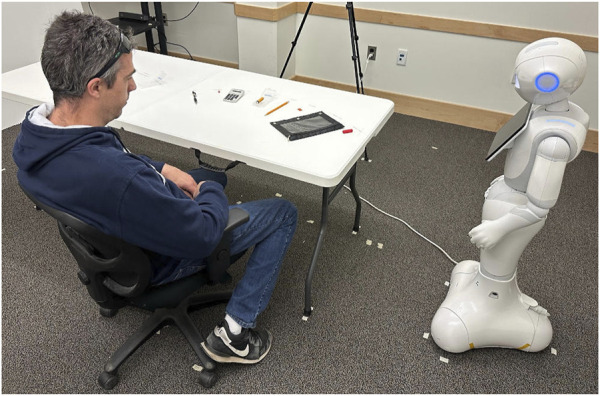
In a human-subjects study, a Pepper robot is about to instruct a human (an experimenter) to assort school supplies using utterances ordered by cognitive statuses of physical objects: “Take the batteries out of the battery box.”

This second, “easy” task involves sorting an assortment of school supplies, such as placing a pencil inside a pencil case. This task is both easy and intuitive, as it involves everyday objects and actions which participants have likely taken many times. The required parts are a pencil, a pencil grip, an eraser, a pen, a pencil case, a calculator, a battery, a battery box, and a sticker. Figure 1 shows the final sorted set of school supplies on the right. As shown in Figure 2, the objects in this task are placed on the table so they are equally accessible within arm’s reach. The second column of Table 2 shows the planner output for this task. The planner outputs and surface realizations, with cognitive statuses, of both models under both tasks are included in supplementary materials.

**TABLE 2:** School supply sorting task plan and instructions.

Planner type	Planner output	Surface realization with cognitive statuses
Classical	(attach pencil-grip pencil)	*“Attach the pencil grip (U) to the pencil (U)”*
	(take-out battery battery-box)	*“Take the battery (U) out of the battery box (U)”*
	(put-in battery calculator)	*“Put it (I) in the calculator (U)”*
	(attach eraser pencil)	*“Attach the eraser (U) to that pencil (F)”*
	(put-in pencil pencil-case)	*“Put that pencil (F) in the pencil case (U)”*
	(put-in pen pencil-case)	*“Put the pen (U) in that (A)”*
	(attach sticker calculator)	*“Attach the sticker (U) to that calculator (A)”*
GH-Informed	(attach sticker calculator)	*“Attach the sticker (U) to the calculator (U)”*
	(take-out battery battery-box)	*“Take the battery (U) out of the battery box (U)”*
	(put-in battery calculator)	*“Put it (I) in that (A)”*
	(attach eraser pencil)	*“Attach the eraser (U) to the pencil (U)”*
	(attach pencil-grip pencil)	*“Attach the pencil grip (U) to that (A)”*
	(put-in pen pencil-case)	*“Put the pen (U) in the pencil case (U)”*
	(put-in pencil pencil-case)	*“Put that (A) in that (A)”*

### 3.3 Cognitive status-referring form mapping

To convert the planner output to spoken instructions, we use a simplified version of the criteria specified in a well-known GH coding protocol (Gundel et al., 2006), as shown in Table 3. However, this led to production of ambiguous referring forms that could have been interpreted as referring to multiple objects that hold the same cognitive statuses.

**TABLE 3:** Coding protocol for cognitive status.

Cognitive status	Criteria	Referring forms
In Focus (I)	Topic-mentioned, last utterance	it, them
Activated (A)	Mentioned, last two utterances	that, those
Familiar (F)	Mentioned	that N, those N
Uniquely ID’able (U)	Not mentioned	the N

As the goal of this work is to evaluate the document planner, not the referring form selection algorithms that may produce ambiguous referring forms, we applied the following common sense reasoning principles to ensure disambiguating referring forms would be used across all conditions. First, when two or more objects are activated or higher (i.e., In-Focus) and cannot be disambiguated based on affordable actions, *that/those* and *it/them* should not be used to refer to one of those objects and the referring form for Familiar cognitive status should be used. Second, if only one object affords a given action, the referring form should not change.

### 3.4 Experimental design

To compare the cognitive status-informed approach with the classical planner, we followed a 2 (document planner type) × 2 (task type) mixed design, with *Task* as a between-subjects factor and *Document Planner Type* as a within-subjects factor. We chose to have Task as a between-subjects factor because finishing a particular task will confound the performance and perception of the other planner finishing the same task. The ordering of each participant’s sequence of two tasks was counterbalanced.

Objective Measures: *Effectiveness* was calculated as success rates for whole tasks and individual instructions. *Instruction Success Rate* was calculated as the percentage that the participants correctly finishes a particular step in the task after following an instruction by a robot. *Task Success Rate* was calculated as the percentage that the participants correctly finish the whole task. *Efficiency* was measured by completion times for whole tasks and individual instructions. *Task Completion Time* was calculated as the difference between the time when the robot starts saying that it has finished all instructions and the time when the robot finishes saying the first instruction of the next task, or that there are no tasks remaining. *Instruction Completion Time* was calculated as the difference between the time when the robot started saying the first instruction and the time when the robot started saying the second instruction, averaged across all seven instructions for each participant. Software recorded both metrics.

Subjective Measures: *Naturalness*, *fluency*, and *understandability* were measured after each within-subjects experimental block using five-point Likert Items, in which participants were asked to indicate how natural, fluent, and understandable Pepper’s instructions had been during the preceding block. *Intelligence* was measured using the Godspeed Perceived Intelligence scale (Bartneck et al., 2009). *Workload* was measured by the NASA Task Load Index (Hart, 2006; NASA, 2019), including its weighting survey components.

### 3.5 Procedure

Participants first filled out an informed consent and, once signed, completed a demographic survey. They then entered an experiment room and sat in front one of a table where their first task was laid out for them. Before starting working on the task, the experimenter got the participants familiar with the task objects and actions like attachment. The experimenter then began the experiment. During the tasks, the participants were asked to say OK to the robot when they were ready for the next instruction. The experimenter sat in another room and manually controlled the robot via Wizard-of-Oz (Riek, 2012) to speak the next instruction after hearing “OK”. This was intentional to not introduce extra time to task completion time for speech recognition errors. When a task is finished, the robot said “I have finished all instructions for this task” and participants were asked to finish a questionnaire containing the subjective measures and the workload survey.

### 3.6 Participants

82 participants’ ages ranged from 18 to 59 (M = 24, SD = 7.5). 39 self-reported as women and 38 as men, with four reporting as gender non-conforming. 38 (46.9%) reported experience with robots, 31 (37.8%) reported no experience, and 12 (14.8%) were neutral. Participants spent 24 min on average to finish the whole experiment and were paid $15 USD. This study was approved by a Human Subjects Research committee.

### 3.7 Data analysis

We conducted a Bayesian statistical analysis (Wagenmakers et al., 2018) using R 4.3.2 and JASP 0.17.2.1 (JASP Team, 2023). Bayesian hypothesis testing allows quantifying evidence for competing hypotheses ( $H_{0}$ vs $H_{1}$ ) as well as $H_{0}$ , using Bayes factor (BF), which is a ratio of likelihood of given data being observed under each of two competing hypotheses. For example, $BF_{10}=5$ indicates that the data are in favor of $H_{1}$ , five times more likely under $H_{1}$ than under $H_{0}$ . The Bayesian framework also allows a flexible sampling plan. Under a Frequentist approach, one needs to conduct a power analysis to identify the sample size needed (Button et al., 2013; Bartlett et al., 2022). The experimenter then needs to run this many participants, and is not permitted to stop early or continue beyond that estimate without violating the underlying assumptions of Frequentist statistical tests. In contrast, the Bayesian approach is not grounded in the central limit theorem and does not strictly require power analyses (Correll et al., 2020). Instead, the experimenter is permitted and encouraged to continue collecting data until enough evidence has been collected to make a claim in favor of one of the competing hypotheses under consideration or until resources have been exhausted. For a comprehensive introduction to the Bayesian approach and its benefits, we refer readers to Wagenmakers et al. (2018).

To interpret Bayes factors, we used the discrete classification scheme that is widely accepted and proposed by Lee and Wagenmakers (2014). For evidence favoring $H_{1}$ , $BF_{10}$ is anecdotal for BF∈(1,3] , moderate for BF∈(3,10] , strong for BF∈(10,30] , very strong for BF∈(30,100] , and extreme for BF∈(100,∞] . For data in favor of $H_{0}$ , these thresholds are inverted (1, 1/3, 1/10, 1/30, 1/100). Anecdotal evidence is deemed as inconclusive for an effect and more data would need to be collected to fully rule in or rule out such effect.

## 4 Results

Effectiveness: For success rate of tasks, 26.8% (22/82) and 29.3% (24/82) participants successfully finished their tasks under the GH-informed document planner and the classical planner, respectively. We ran a Bayesian binomial test to see whether there was a difference in task success rate. The tests revealed moderate evidence against such difference $\left(BF_{10}=0.18\right)$ . Thus, H1.1 was not supported. Participants were equally likely to finish their tasks with instructions from each planner. For success rate of instructions, 94.1% (540/574) and 92.5% (531/574) of individual instructions were successfully completed under the GH-informed document planer and the classical planner, respectively. A Bayesian binomial test revealed moderate evidence against a difference between planner types $\left(BF_{10}=0.28\right)$ . Thus, H1.2 was also not supported. Participants were overall equally likely to complete instructions at the individual level with instructions from each planner, although the *mean* instruction-level success rate was slightly higher for the GH-informed planner.

**FIGURE 3:**
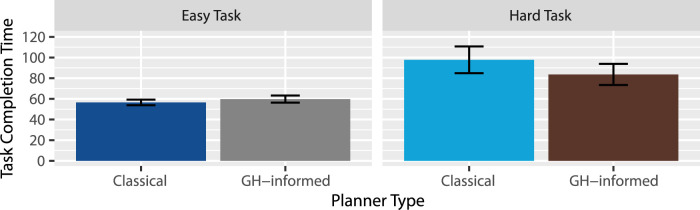
Average task completion time (seconds).

Efficiency: A two-way repeated measures Analysis of Variance (RM-ANOVA) revealed extreme evidence favoring an effect of task $\left(BF_{10}=2.32\times 10^{11}\right)$ with participants completing the easy task significantly faster (M=58.15 s) than the hard task (M=90.71 s) . Anecdotal evidence was found against an effect of planner $\left(BF_{10}=0.43\right)$ , suggesting there is probably no such effect, and if there is, it would be that tasks took longer to complete when sequenced by the classical planner (M=77.15 s) than by the GH-informed planner (M=71.71 s) . Anecdotal evidence was found in favor of an interaction between task type and planner type $\left(BF_{10}=1.10\right)$ . As shown in Figure 3, it may be that the GH-informed planner outperformed the classical planner within the hard task. This suggests that H2 is partially supported: People may have finished tasks faster under the GH-informed planner than under the classical planner, but only for the hard task.

**FIGURE 4:**
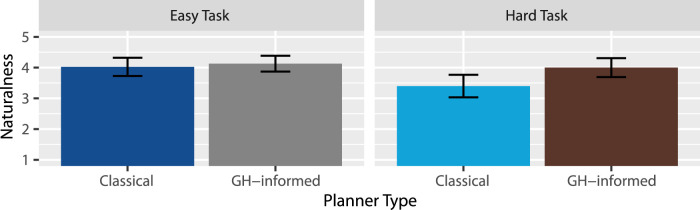
Mean naturalness ratings.

Naturalness: A two-way RM-ANOVA revealed extreme evidence for an effect of task type $\left(BF_{10}=146.86\right)$ with the robot’s instructions in the hard task being less natural $\left(M_{\mathrm{hard}}=3.70\right)$ than in the easy task $\left(M_{\mathrm{easy}}=4.08\right)$ . Very strong evidence was found for an effect of planner type $\left(BF_{10}=95.58\right)$ , suggesting that the robot’s instructions were less natural when generated by the classical planner $\left(M_{\mathrm{classical}}=3.71\right)$ than by the GH-informed planner $\left(M_{GH}=4.06\right)$ . Finally, the RM-ANOVA revealed anecdotal evidence against an interaction between task type and planner type $\left(BF_{10}=0.46\right)$ . This suggests there is probably no interaction, but as seen in Figure 4, if there is an effect, it would be that the GH-informed planner outperformed the classical planner more so within the hard task than in the easy task. Overall, our results suggest that H3 was supported: The instructions under the GH-informed planner were perceived as more natural than under the classical planner.

**FIGURE 5:**
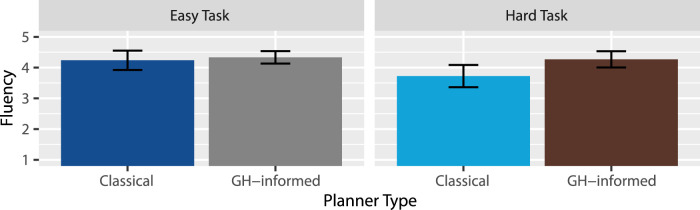
Mean fluency ratings.

Fluency: A two-way RM-ANOVA revealed anecdotal evidence in favor of an effect of task type $\left(BF_{10}=2.34\right)$ , with the robot’s instructions in the hard task being less fluent $\left(M_{\mathrm{hard}}=4.00\right)$ than in the easy task $\left(M_{\mathrm{easy}}=4.29\right)$ . Moderate evidence was found in favor of an effect of planner $\left(BF_{10}=5.69\right)$ , suggesting that the robot’s instructions were less natural when generated by the classical planner $\left(M_{\mathrm{classical}}=3.98\right)$ than by the GH-informed planner $\left(M_{GH}=4.3\right)$ . Finally, the RM-ANOVA revealed anecdotal evidence against an interaction $\left(BF_{10}=0.69\right)$ . This suggests there is probably no interaction, but as seen in Figure 5, if there is an effect, it would be that the GH-informed planner outperformed the classical planner more so within the hard task than in the easy task. Overall, our results suggest that H4 was supported: The instructions under the GH-informed planner were perceived as more fluent than under the classical planner.

**FIGURE 6:**
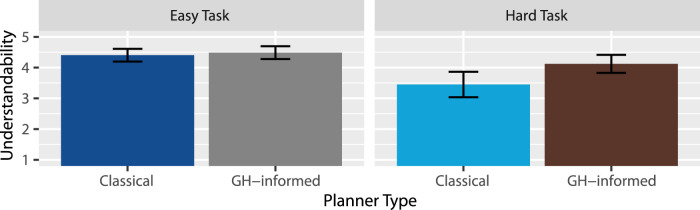
Mean understandability ratings.

Understandability: A two-way RM-ANOVA revealed extreme evidence for an effect of task type $\left(BF_{10}=3.61\times 10^{4}\right)$ with the robot’s instructions in the hard task being less understandable (M=3.79) than in the easy task (M=4.45) strong evidence was found in favor of an effect of planner type $\left(BF_{10}=11.34\right)$ , with the robot’s instructions in the classical planner condition being less understandable (M=3.93) than in the GH-informed condition (M=4.30) . Finally, the RM-ANOVA revealed anecdotal evidence against an interaction between task type and planner type $\left(BF_{10}=0.74\right)$ , suggesting there is probably no such effect, but as seen in Figure 6, if there is an effect, it would be that the GH-informed planner outperformed the classical planner more so within the hard task than in the easy task. Thus, H5 was supported: The instructions under the GH-informed planner were perceived as more understandable than under the classical planner.

**FIGURE 7:**
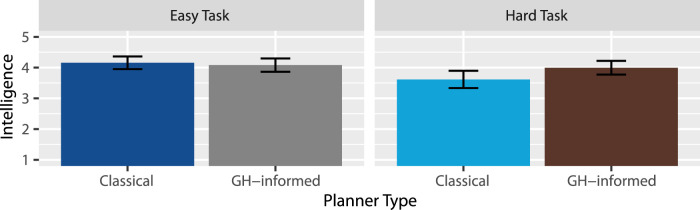
Mean intelligence ratings.

Perceived Intelligence: A two-way RM-ANOVA revealed extreme evidence in favor of an effect of task type $\left(BF_{10}=3.53\times 10^{3}\right)$ with the robot’s instructions in the hard task perceived less intelligent (M=3.81) than in the easy task (M=4.12) . Moderate evidence was found in favor of an effect of planner $\left(BF_{10}=5.78\right)$ , with the robot’s instructions in the classical planner condition (M=3.89) being less intelligent than in the GH-informed condition (M=4.03) . Finally, the RM-ANOVA revealed anecdotal evidence against an interaction between task type and planner type $\left(BF_{10}=0.52\right)$ , suggesting there is probably no such effect, but as seen in Figure 7, if there is an effect, it would be that the GH-informed planner outperformed the classical planner more so within the hard task than in the easy task. Thus, H6 was supported: The robot was perceived as intelligent given the human cognitive modeling of object statuses.

**FIGURE 8:**
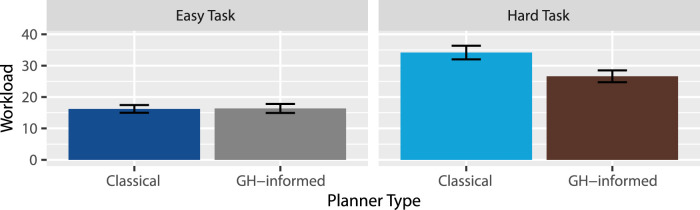
Mean workload ratings.

Workload: According to the NASA Task Load Index manual (NASA, 2019), a weighted score was calculated for each participant, the results of which are summarized in Figure 8. A two-way RM-ANOVA revealed extreme evidence favoring an effect of task $\left(BF_{10}=1.29\times 10^{9}\right)$ with the robot’s instructions in the hard task requiring more workload (M=30.45) than in the easy task (M=16.20) . Anecdotal evidence was found in favor of an effect of planner $\left(BF_{10}=1.05\right)$ , suggesting that the instructions given by the classical planner (M=25.10) may have required more workload than those given by the GH-informed planner (M=21.54) . Finally, the RM-ANOVA revealed anecdotal evidence against an interaction between task type and planner type $\left(BF_{10}=0.66\right)$ , suggesting there is probably no such effect, but as seen in Figure 8, if there is an effect, it would be that the GH-informed planner outperformed the classical planner more so within the hard task than in the easy task. Thus, H7 was partially supported: Following the instructions provided by the GH-informed planner may have required less workload, but only for the hard task.

## 5 Discussion

Hypothesis 1 (H1) - Increased Effectiveness proposes that more people will successfully finish the task (H1.1) and at each step (H1.2) under the GH-informed planner. Our results do not support this, suggesting that the planner type had little-to-no effect on the completion rate of tasks and instructions. The variance in instruction and task completion rates among individuals is likely due to a variety of factors, with the planner type playing a small role, if any. The length of instructions, and therefore the time it took for the robot to utter the instructions, were close between planner types, so there was no significant time lost due to longwindedness. Sample size may have limited this conclusion, as instructions may have been interpreted differently among age groups or education levels, in ways not assessable with our sample size. The complexity of the tasks may have also been not well suited to measure variance in completion times. Both tasks, across both planner types, could be completed with seven instructions, all of which could be phrased directly, concisely, and with little ambiguity. By experimental design, participants did not often have to decipher ambiguity, and were almost always quick to successfully determine what entity is being referred to. Future work could investigate introducing ambiguity, and could examine whether participants spent time deliberating the meaning of ambiguous referents.

Hypothesis 2 (H2) - Increased Efficiency proposes that people will finish tasks faster (H2.1) and finish each step faster (H2.2) under the GH-informed planner. Our results partially support this hypothesis, suggesting that the difference in efficiency as a result of the GH-informed planner may only arise in complex tasks. It may be the case that the GH-informed planner excels with more complex problems, or that the easy task was so simple that participants were able to guess the correct completion of an instruction, regardless of what the robot uttered. Future experiments should compare these planner types with more complex tasks, either by adding more steps or by using a more difficult problem.

Hypothesis 3 (H3) - Higher Perceived Naturalness proposes that the instructions will be perceived as more natural under the GH-informed planner. Our results support this claim. From this, we gather that the GH-informed planner can produce sequences of utterances that sound more natural. Future work may consider more complex or longer instructions, or possibly a set of utterances that may be difficult to string together in a natural-sounding way.

Hypothesis 4 (H4) - Increased Fluency proposes that the instructions will be perceived as more fluent under the GH-informed planner. Our results support it. From this, we gather that the GH-informed planner can produce sequences of utterances that sound more fluent. Future work may consider more complex or longer instructions, or possibly a set of utterances that may be difficult to string together fluently.

Hypothesis 5 (H5) - Increased Understandability proposes that the instructions will be rated more understandable under the GH-informed planner. Our results support this claim, suggesting that the robot’s instructions were rated as more understandable in the GH-informed cases. From this, we gather that the GH-informed planner is able to produce sequences of utterances that are more understandable, and is thus better suited for natural language generation in contexts where understandability is important. Future work may consider more complex or longer instructions, or possibly a set of utterances that may be difficult to string together in an understandable way.

Hypothesis 6 (H6) - Higher Perceived Intelligence proposes that the robot will be perceived as more intelligent given the human cognitive modeling of object statuses under the GH-informed planner. Our results support this claim. From this, we gather that the GH-informed planner can produce sequences of utterances that make the robot sound more intelligent. Future work may consider more complex or longer instructions, or possibly a set of utterances that may be difficult to string together intelligently.

Hypothesis 7 (H7) - Lower Workload proposes that following these instructions will require less workload under the GH-informed planner. Our results partially supported this claim, suggesting that the GH-informed planner was able to produce instructions that were easier to follow in more complex tasks. Similar to H2, the GH-informed planner may be better suited for more complex tasks. Likewise, it may be that the easy task in our experiment was too simple and did not require participants to exert a significant enough mental demand. As with H2, future work should include more complex tasks that demand higher workload from participants.

### 5.1 Limitations and future work

First, we have hand-coded common-sense reasoning rules as described above. Ideally these principles would be encoded algorithmically. However, we believe our hand-application of these rules is reasonable in this work given that REG is not the focus of this work. Second, the referring expressions used may not be the optimal referring expressions for disambiguation. Again, however, we believe this is a reasonable limitation given that we did not focus on REG in this work. Third, within our study, we only assessed two unique tasks, and had only one instruction set for each combination of task and planner type. The initial findings from this work should be replicated in future work across a wider array of tasks of varying difficulties, and with a varied set of instructions within each task. It should also be noted that our sample size (N=82) consisted of primarily college-aged students. Future work should replicate this work with a wider array of participants. Fourth, we believe adding gestures using arms may improve the success rate, which future work should investigate such multimodality with comparisons with unimodal smart speakers. Finally, in this experiment, each participant saw both planner types and both task types, but never all four combinations of planner and task types. This limited our analyses, as it was not possible to analyze a specific participant’s experience of both planner types for the same task, or both tasks under the same planner type.

## 6 Conclusion

In this work, we have leveraged GH to enable robots to plan their utterances in a way that keeps objects at a high cognitive status. We conducted a human-subjects study to compare our GH-informed planner with a classical baseline. Our results showed that, on average, the GH-informed planner outperforms the baseline classical planner, with participants rating the GH-informed planner as more natural, fluent, understandable, and intelligent, as well as completing tasks in the GH-informed experiment at a faster rate and with less cognitive workload. This suggests a clear opportunity for the use of GH-informed planning to enable more effective robot-driven instruction, both in training contexts like those explored in this work, and in socially assistive robotics contexts such as robot-assisted tutoring.

## Data Availability

The datasets presented in this study can be found in online repositories. The names of the repository/repositories and accession number(s) can be found below: https://osf.io/9yfzx.

## References

[B1] Arnold J. E. Zerkle S. A. (2019). Why do people produce pronouns? Pragmatic selection vs. rational models. Lang. Cognition Neurosci. 34, 1152–1175. 10.1080/23273798.2019.1636103

[B2] Bartlett M. E. Edmunds C. Belpaeme T. Thill S. (2022). Have I got the power? Analysing and reporting statistical power in HRI. ACM Trans. Human-Robot Interact. (THRI) 11, 1–16. 10.1145/3495246

[B3] Bartneck C. Kulić D. Croft E. Zoghbi S. (2009). Measurement instruments for the anthropomorphism, animacy, likeability, perceived intelligence, and perceived safety of robots. Int. J. Soc. robotics 1, 71–81. 10.1007/s12369-008-0001-3

[B4] Bennett M. Williams T. Thames D. Scheutz M. (2017). Differences in interaction patterns and perception for teleoperated and autonomous humanoid robots. IEEE/RSJ International Conference on Intelligent Robots and Systems IROS, 6589–6594.

[B5] Button K. S. Ioannidis J. Mokrysz C. Nosek B. A. Flint J. Robinson E. S. (2013). Power failure: why small sample size undermines the reliability of neuroscience. Nat. Rev. Neurosci. 14, 365–376. 10.1038/nrn3475 23571845

[B6] Campana E. Tanenhaus M. K. Allen J. F. Remington R. (2011). Natural discourse reference generation reduces cognitive load in spoken systems. Nat. Lang. Eng. 17, 311–329. 10.1017/s1351324910000227 25328423 PMC4199659

[B7] Chai J. Y. Hong P. Zhou M. X. (2004). “A probabilistic approach to reference resolution in multimodal user interfaces,” in Proceedings of the 9th international conference on intelligent user interfaces, 70–77.

[B8] Correll J. Mellinger C. McClelland G. H. Judd C. M. (2020). Avoid cohen’s ‘small’,‘medium’, and ‘large’for power analysis. Trends Cognitive Sci. 24, 200–207. 10.1016/j.tics.2019.12.009 31954629

[B9] Fang R. Liu C. She L. Chai J. (2013). “Towards situated dialogue: revisiting referring expression generation,” in Proceedings of the 2013 conference on empirical methods in natural language processing, 392–402.

[B10] Gatt A. Krahmer E. (2018). Survey of the state of the art in natural language generation: core tasks, applications and evaluation. J. Artif. Intell. Res. 61, 65–170. 10.1613/jair.5477

[B11] Grice H. P. (1975). “Logic and conversation,” in Speech acts (Leiden, Netherlands: Brill), 41–58.

[B12] Gundel J. K. Hedberg N. Zacharski R. (1993). Cognitive status and the form of referring expressions in discourse. Language 69, 274–307. 10.2307/416535

[B13] Gundel J. K. Hedberg N. Zacharski R. Mulkern A. Custis T. Swierzbin B. (2006). Coding protocol for statuses on the givenness hierarchy.

[B14] Gundel J. K. Bassene M. Gordon B. Humnick L. Khalfaoui A. (2010). Testing predictions of the givenness hierarchy framework: a crosslinguistic investigation. J. Pragmat. 42, 1770–1785. 10.1016/j.pragma.2009.09.010

[B15] Hadar B. Skrzypek J. E. Wingfield A. Ben-David B. M. (2016). Working memory load affects processing time in spoken word recognition: evidence from eye-movements. Front. Neurosci. 10, 221. 10.3389/fnins.2016.00221 27242424 PMC4871876

[B16] Han Z. Williams T. (2022). “A task design for studying referring behaviors for linguistic HRI,” in *2022 17th ACM/IEEE international conference on human-robot interaction (HRI)* (IEEE), 783–786.

[B17] Han Z. Norton A. McCann E. Baraniecki L. Ober W. Shane D. (2021). Investigation of multiple resource theory design principles on robot teleoperation and workload management. IEEE International Conference on Robotics and Automation ICRA, 3858–3864.

[B18] Han Z. Rygina P. Williams T. (2022). “Evaluating referring form selection models in partially-known environments,” in Proceedings of the 15th international conference on natural Language generation, 1–14.

[B19] Hart S. G. (2006). “Nasa-task load index (NASA-TLX); 20 years later,”, 50. Los Angeles, CA: Sage publications Sage, 904–908. 10.1177/154193120605000909

[B20] Higger M. Williams T. (2024). “GAIA: a givenness hierarchy theoretic model of situated referring expression generation,” in Annual meeting of the cognitive science society.

[B21] JASP Team (2023). JASP (Version 0.17.2.1)[Computer software].

[B22] Kehler A. (2000). “Cognitive status and form of reference in multimodal human-computer interaction,” in Proceedings of the seventeenth national conference on artificial intelligence and twelfth conference on innovative applications of artificial intelligence (AAAI Press), 685–690.

[B23] Krahmer E. Van Deemter K. (2012). Computational generation of referring expressions: a survey. Comput. Linguist. 38, 173–218. 10.1162/coli_a_00088

[B24] Lee M. D. Wagenmakers E.-J. (2014). Bayesian cognitive modeling: a practical course. Cambridge, United Kingdom: Cambridge University Press.

[B25] Levinson S. C. (2004). “Deixis,” in The handbook of pragmatics (Oxford, United Kingdom : Blackwell), 97–121.

[B26] McDonald D. D. (1993). Issues in the choice of a source for natural language generation. Comput. Linguist. 19, 191–197. 10.5555/972450.972461

[B27] Meo T. McMahan B. Stone M. (2014). “Generating and resolving vague color references,” in Proceedings of the 18th workshop on the semantics and pragmatics of dialogue (SemDial), 107–115.

[B28] NASA (2019). NASA TLX paper and pencil version. Available online at: https://humansystems.arc.nasa.gov/groups/tlx/tlxpaperpencil.php (Accessed May 09, 2022).

[B29] Pal P. Zhu L. Golden-Lasher A. Swaminathan A. Williams T. (2020). “Givenness hierarchy theoretic cognitive status filtering,” in Proceedings of the annual meeting of the cognitive science society.

[B30] Pal P. Clark G. Williams T. (2021). “Givenness hierarchy theoretic referential choice in situated contexts,” in Proceedings of the Annual Meeting of the Cognitive Science Society.

[B31] Reiter E. (2000). Pipelines and size constraints. Comput. Linguist. 26, 251–259. 10.1162/089120100561692

[B32] Reiter E. Dale R. (1997). Building applied natural language generation systems. Nat. Lang. Eng. 3, 57–87. 10.1017/s1351324997001502

[B33] Riek L. D. (2012). Wizard of oz studies in hri: a systematic review and new reporting guidelines. J. Human-Robot Interact. 1, 119–136. 10.5898/jhri.1.1.riek

[B34] Roy D. K. (2002). Learning visually grounded words and syntax for a scene description task. Comput. speech and Lang. 16, 353–385. 10.1016/s0885-2308(02)00024-4

[B35] Spevak K. Han Z. Williams T. Dantam N. T. (2022). Givenness hierarchy informed optimal document planning for situated human-robot interaction. IEEE/RSJ International Conference on Intelligent Robots and Systems IROS, 6109–6115.

[B36] Tily H. Piantadosi S. (2009). “Refer efficiently: use less informative expressions for more predictable meanings,” in Proceedings of the workshop on the production of referring expressions: bridging the gap between computational and empirical approaches to reference.

[B37] Wagenmakers E.-J. Marsman M. Jamil T. Ly A. Verhagen J. Love J. (2018). Bayesian inference for psychology. Part I: theoretical advantages and practical ramifications. Psychonomic Bull. and Rev. 25, 35–57. 10.3758/s13423-017-1343-3 28779455 PMC5862936

[B38] Wallbridge C. D. Smith A. Giuliani M. Melhuish C. Belpaeme T. Lemaignan S. (2021). The effectiveness of dynamically processed incremental descriptions in human robot interaction. ACM Trans. Human-Robot Interact. (THRI) 11, 1–24. 10.1145/3481628

[B39] Williams T. Scheutz M. (2019). Reference in robotics: a givenness hierarchy theoretic approach in The Oxford Handbook of Reference (Oxford University Press).

[B40] Williams E. Arnold J. (2019). “Priming discourse structure guides pronoun comprehension,” in Poster, CUNY conference on human sentence processing (University of Colorado).

[B42] Williams T. Scheutz M. (2017). “Referring expression generation under uncertainty: algorithm and evaluation framework,” in Proceedings of the 10th international conference on natural language generation, 75–84.

[B43] Williams T. Acharya S. Schreitter S. Scheutz M. (2016). Situated open world reference resolution for human-robot dialogue. 11th ACM/IEEE Int. Conf. Human-Robot Interact. (HRI), 311–318. 10.1109/HRI.2016.7451767

[B44] Xie B. Salvendy G. (2000). Review and reappraisal of modelling and predicting mental workload in single-and multi-task environments. Work and stress 14, 74–99. 10.1080/026783700417249

[B45] Zarrieß S. Schlangen D. (2016). “Towards generating colour terms for referents in photographs: prefer the expected or the unexpected?,” in Proceedings of the 9th international natural language generation conference, 246–255.

